# Supplementary material for: Transient destabilization of whole brain dynamics induced by N,N-Dimethyltryptamine (DMT)
Source: Commun Biol. 2025 Mar 11;8:409. doi: 10.1038/s42003-025-07576-0 (PMC11897362; doi:10.1038/s42003-025-07576-0)
Supplement: Supplementary file 1 — Supplementary Information [file 42003_2025_7576_MOESM1_ESM.pdf]

Supplementary Material for: "Transient destabilization of whole brain dynamics induced by N,N-Dimethyltryptamine (DMT)"

Juan Ignacio Piccinini[1*], Yonatan Sanz Perl[1,2], Carla Pallavicini[1, 3], Gustavo Deco[2,4,5,6,] Morten Kringelbach[7,8,9], David Nutt[10], Robin Carhart-Harris[10, 11], Christopher Timmermann[10], Enzo Tagliazucchi[1,12*]

[1]Universidad de Buenos Aires, Facultad de Ciencias Exactas y Naturales, Departamento de Física, and CONICET - Universidad de Buenos Aires, Instituto de Física Aplicada e Interdisciplinaria (INFINA). Buenos Aires, Argentina.

[2]Center for Brain and Cognition, Computational Neuroscience Group, Department of Information and Communication Technologies, Universitat Pompeu Fabra, Barcelona, Spain

[3]Integrative Neuroscience and Cognition Center, CNRS, Université Paris Cité, Paris, France.

[4]Institució Catalana de la Recerca i Estudis Avançats (ICREA), Barcelona, Spain

[5]Department of Neuropsychology, Max Planck Institute for Human Cognitive and Brain Sciences, Leipzig, Germany

[6]School of Psychological Sciences, Monash University, Melbourne, Clayton VIC, Australia

[7]Centre for Eudaimonia and Human Flourishing, University of Oxford, Oxford, UK

[8]Department of Psychiatry, University of Oxford, Oxford, UK

[9]Center for Music in the Brain, Department of Clinical Medicine, Aarhus University, Denmark

[10]Centre for Psychedelic Research, Division of Psychiatry, Department of Brain Sciences, Imperial College London, London, UK

[11]Departments of Neurology and Psychiatry, University of California San Francisco, San Francisco, California, USA

[12]Latin American Brain Health Institute (BrainLat), Universidad Adolfo Ibañez, Santiago, Chile

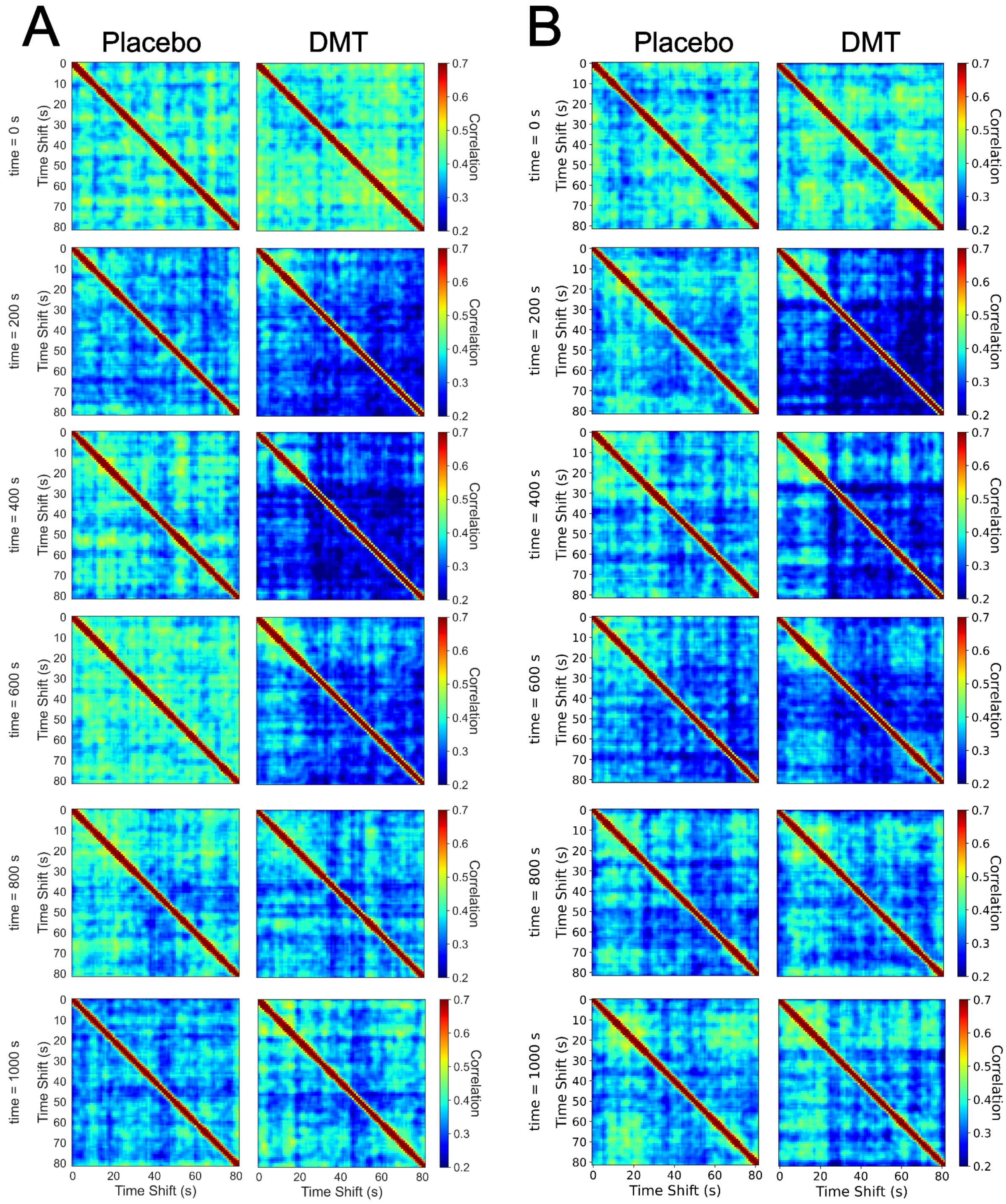

Supplementary Figure 1. Plots of the Functional Connectivity Dynamics (FCD) over time for 6 different time points corresponding to different bifurcation parameters drawn from the optimal gamma function for each condition. Results are shown for DMT and placebo, without perturbation (panel A) and with a perturbation amplitude of 0.01 applied at the visual resting state network (RSN) (panel B) (DMT: dimethyltryptamine).

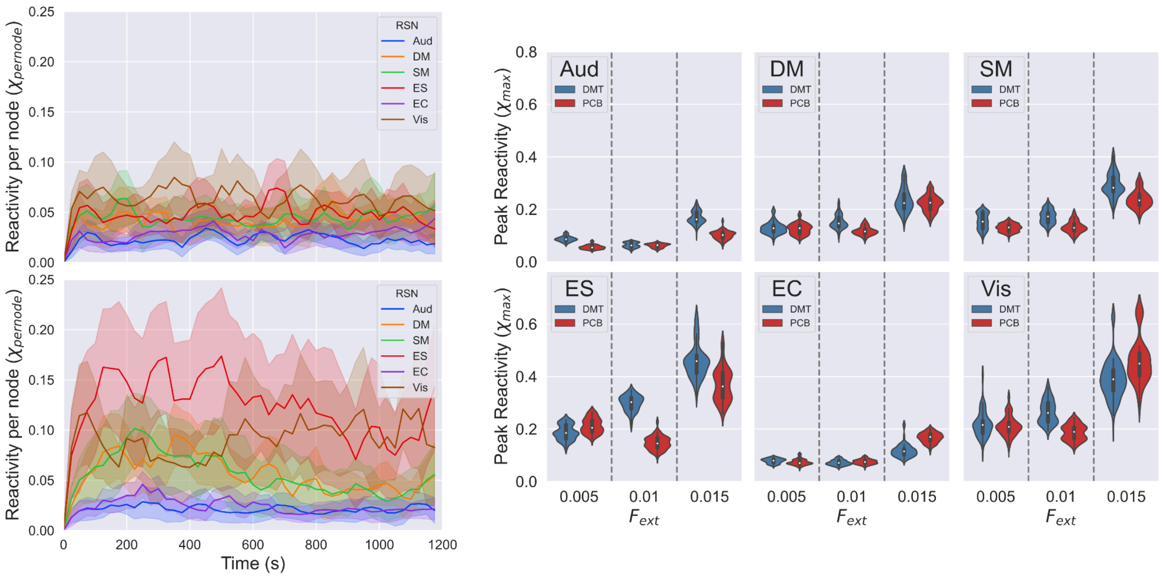

Supplementary Figure 2. Time-dependent effects of simulated perturbations indicate higher reactivity for DMT vs. placebo for inter-network connections. To compute the inter-network FCD, the time series of the nodes corresponding to each network were grouped, and afterwards were averaged to obtain a mean time series per RSN. This resulted in six time series that were used to compute an FCD matrix. Given that the RSNs were perturbed independently, this resulted in six perturbed FCD matrices which were used to calculate the reactivity. In the left panel we show the reactivity normalized by the number of nodes in the corresponding resting state network (RSN), for placebo (top) and DMT (bottom). Shaded regions of each line denote the standard deviation of the reactivities . Left panel depicts the plots of the bootstrapped ($n_{boot} = 200$) peak $\chi(t)$ across time (χ_{max},) for each RSN and three different external perturbation intensities (F_{ext}) (DMT: dimethyltryptamine).

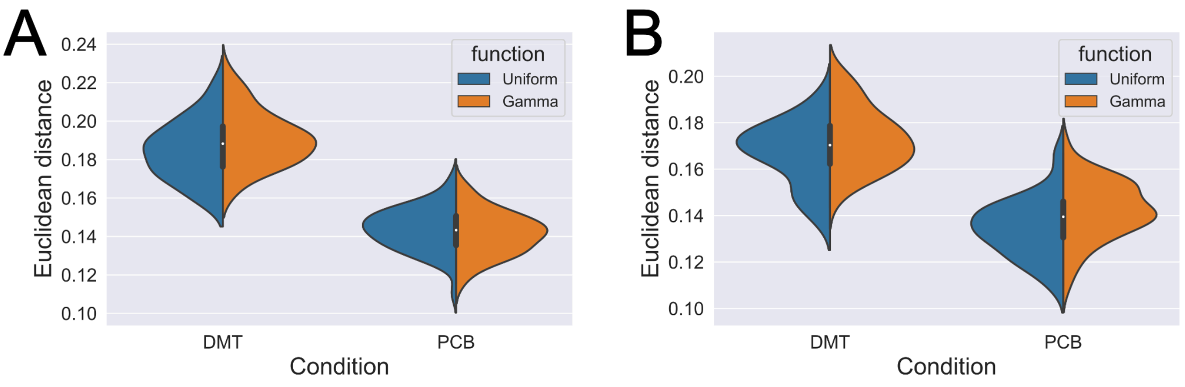

Supplementary Figure 3. The left panel compares the minima for the null-model (uniform function) and the gamma model when analyzing the entire FCD. The right panel compares the minima for the null-model (uniform function) and the gamma model when comparing only the elements of functional connectivity density (FCD) post dose. The Kolmogorov Smirnov test shows no difference between models for the entire FCD in either condition. For the post-dose FCD, the uniform model shows lower values for the placebo (PCB) vs. DMT condition (DMT: dimethyltryptamine).

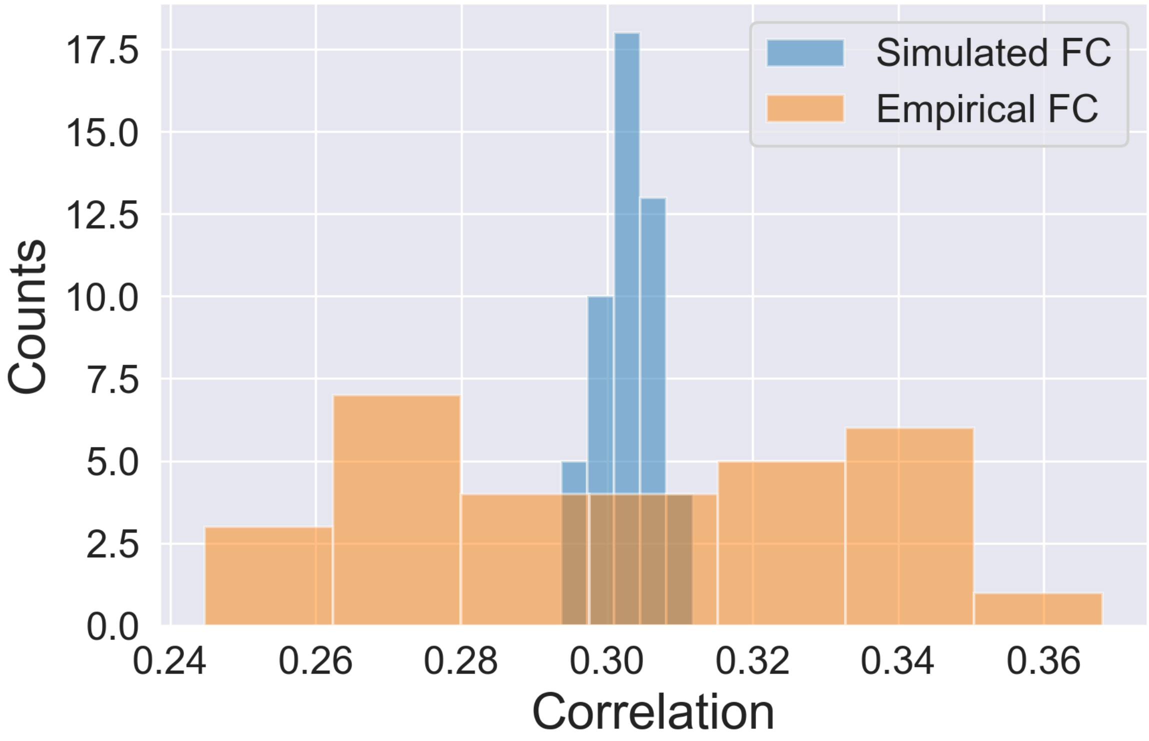

Supplementary Figure 4. Pearson correlation between the simulated Functional Connectivity (FC) and the Structural Connectivity (SC) for the baseline period, i.e. the FC matrices were calculated for the time before the infusion of DMT or placebo. The histograms were obtained across independent simulations of the model and for every subject of the study in each condition. The average correlation between FC and SC is 0.303 ± 0.008 and 0.30 ± 0.06 (95% confidence interval), for the simulations and subjects respectively (DMT: dimethyltryptamine).

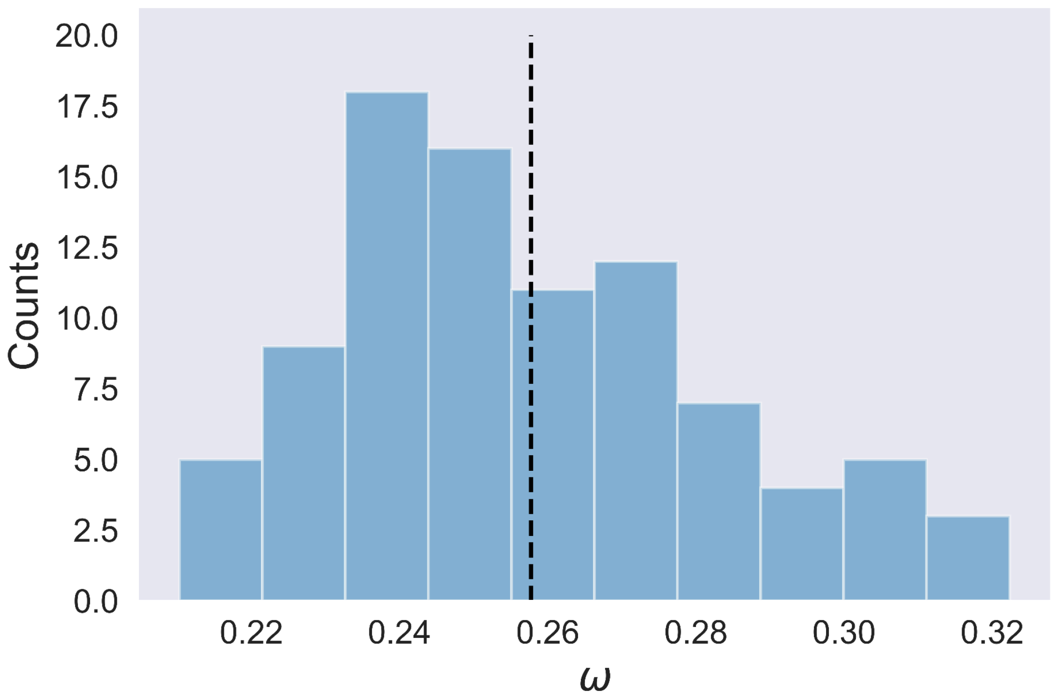

Supplementary Figure 5. Histogram of empirical frequencies of regional fMRI time series that were used as input for the simulations. The dotted vertical line indicates the convergence frequency of the simulated local dynamics at the optimal choice of model parameters, which agrees with the average value of the empirical frequencies (fMRI: functional magnetic imaging).

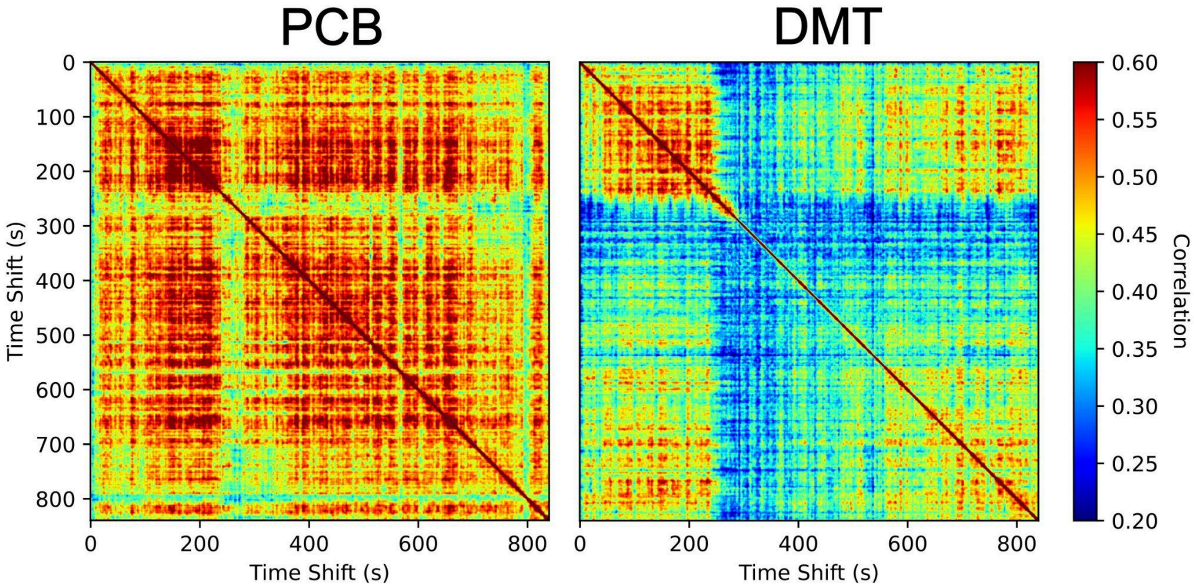

Supplementary Figure 6. Empirical functional connectivity density (FCD) matrices computed using the coherence between fMRI time series. The i,j element of each matrix denotes the correlation between the coherence matrices associated to times i and j, respectively.

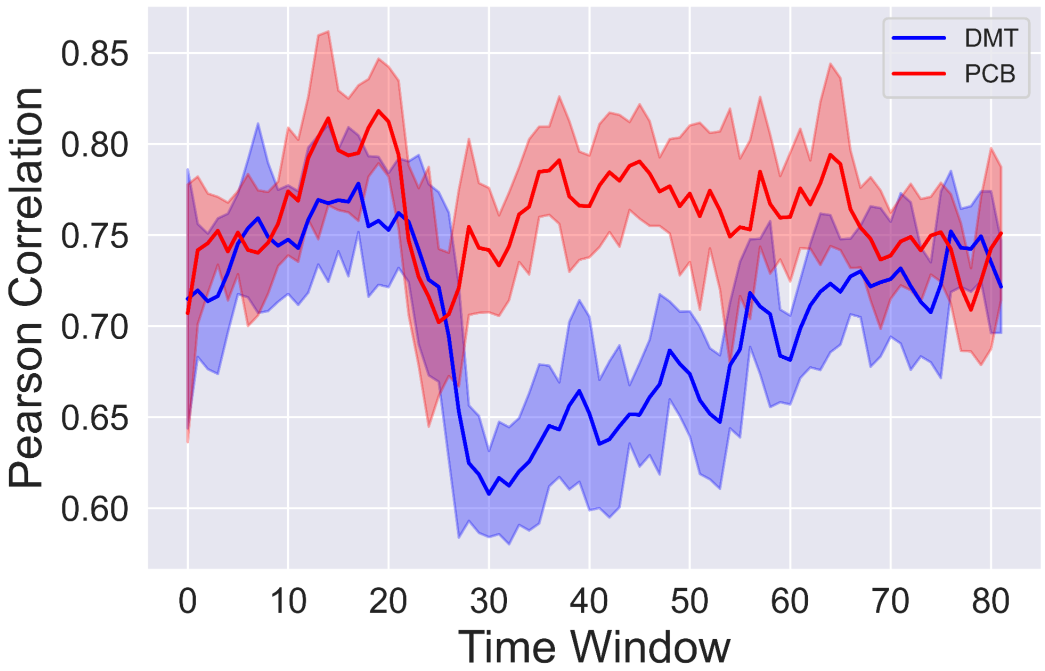

Supplementary Figure 7. Correlation between window-based FC and the coherence empirical matrices. The bold line denotes the mean value of the correlation between the FC matrix and the coherence, computed for every time point within the same temporal window, i.e, each value in the plot is the average between each window-based FC and all the coherence matrices within that time window. Shaded regions indicate the standard deviation from the mean. (PCB: Placebo; DMT: dimethyltryptamine; FC: Functional Connectivity).

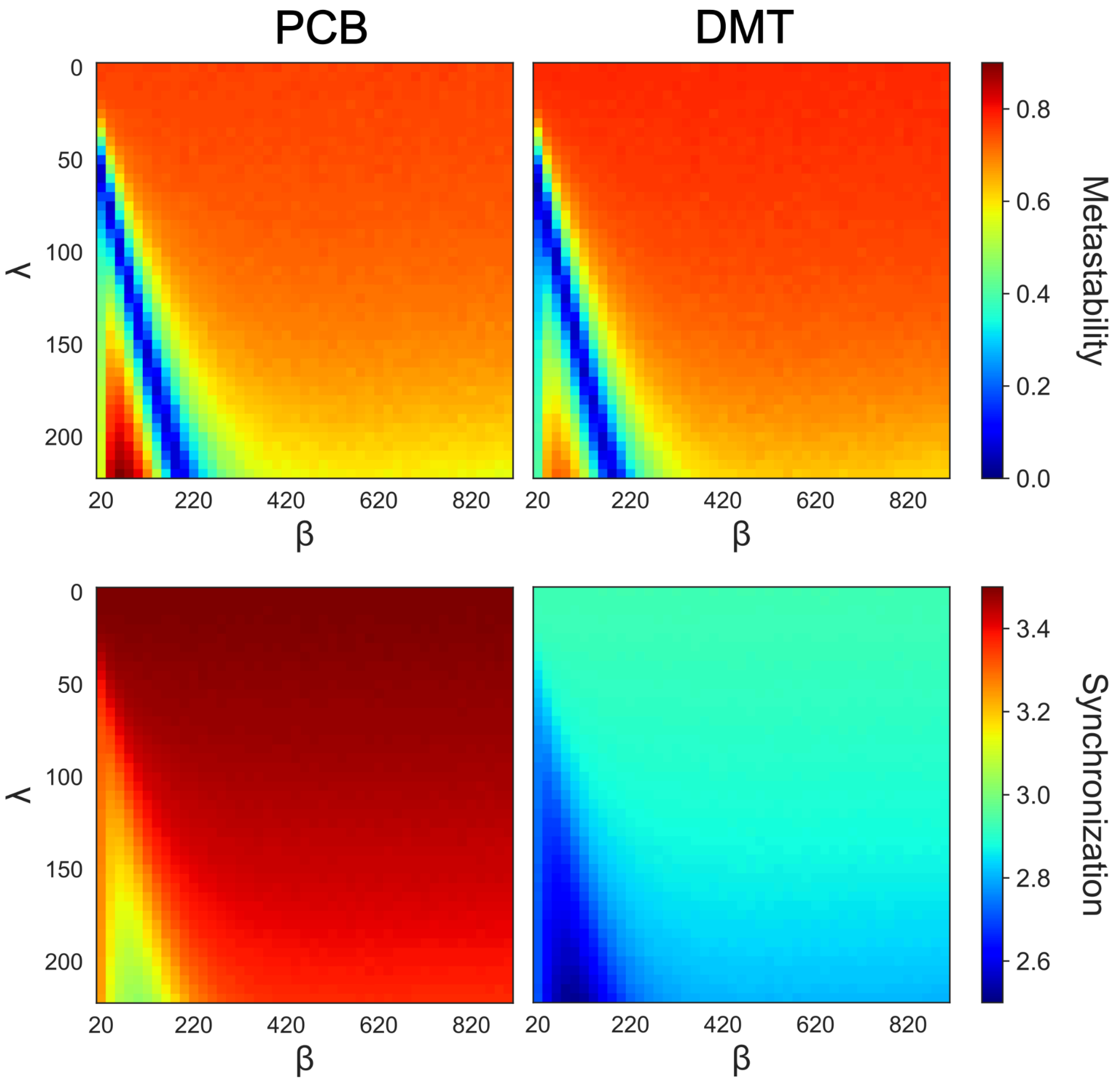

Supplementary Figure 8. Synchronization and metastability between simulated and empirical data averaged over n = 50 independent simulations for every pair of model parameters λ and β. Both metrics were calculated as a relative distance between the corresponding simulated and the empirical observable, i.e., $M = \frac{Osim - Oemp}{Oemp}$, where M represents the distance metric, o_{sim} indicates either the synchrony (time average of Kuramoto order parameter) or metastability (standard deviation of the Kuramoto order parameter) obtained from the simulated data. O_{emp} denotes the corresponding empirical values. (PCB: Placebo; DMT: dimethyltryptamine).

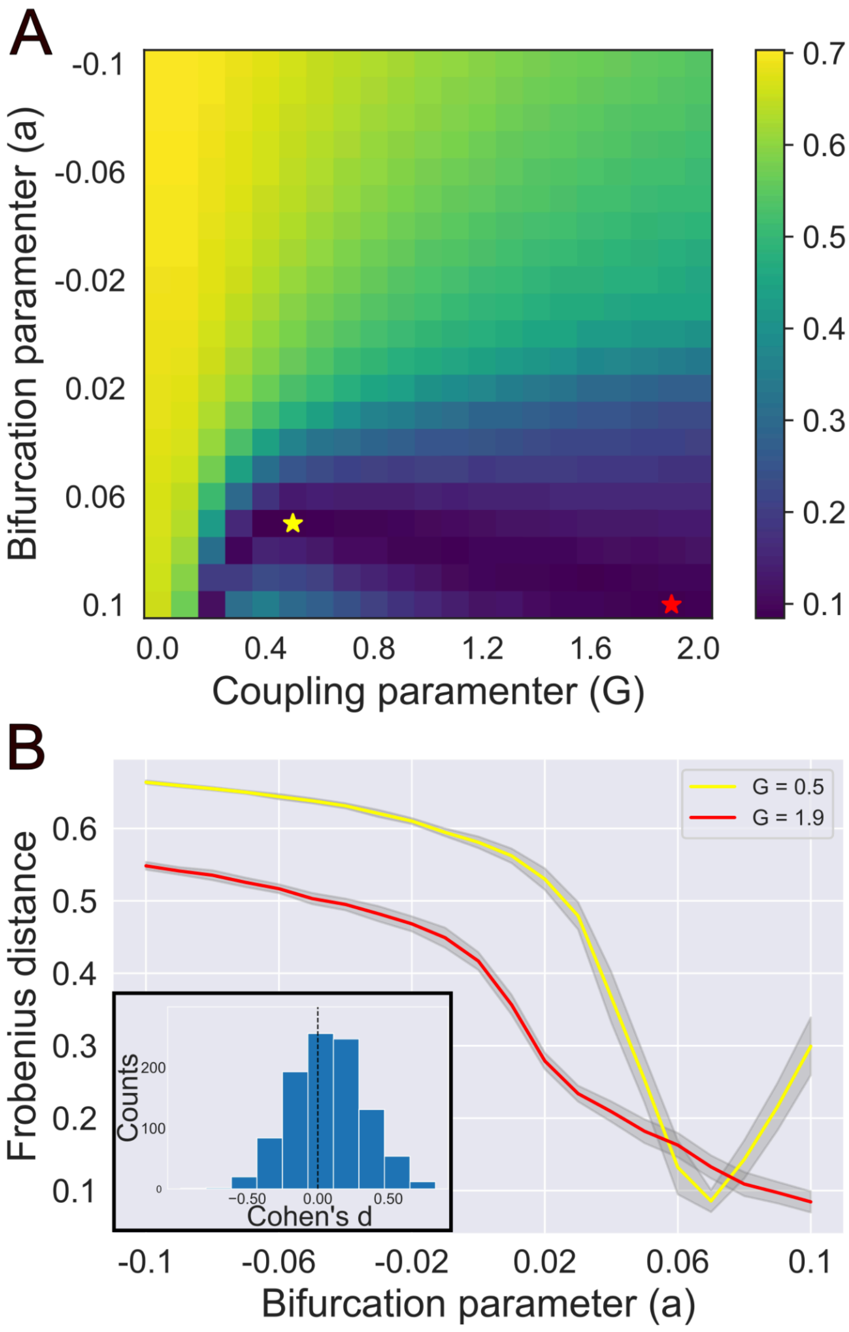

Supplementary Figure 9. (**A)** Exhaustive exploration of the 2D parameter space for the Hopf model applied to the baseline condition (i.e. before DMT infusion), averaged over 30 simulations for each parameter pair. The colorbar indicates the Euclidean distance between the simulated and empirical FCD matrices. The global minimum and the minimum corresponding to G=0.5 are marked with a red and yellow star, respectively. **(B)** Euclidean distance as a function of the bifurcation parameter for the two coupling parameters indicated in panel A. The inset shows the distribution of Cohen's d effect size estimate between both choices of model parameters, obtained using a bootstrap procedure with n = 1000 repetitions. No significant difference in the goodness of fit was found between the two model parameter values (DMT: dimethyltryptamine).

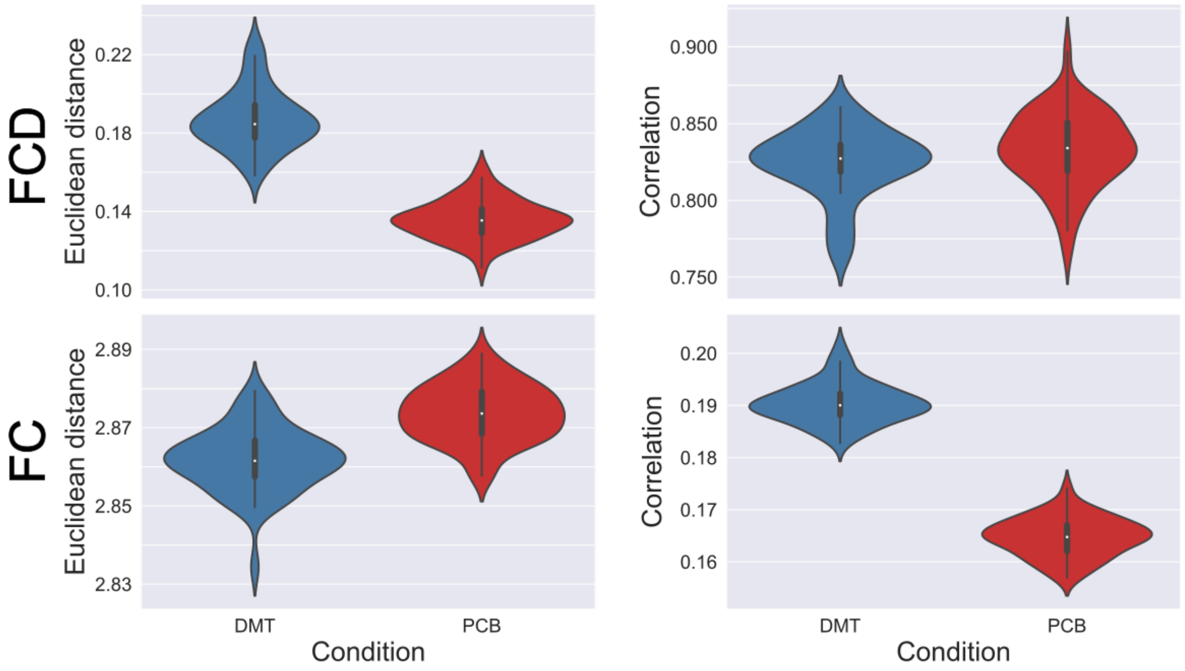

Supplementary Figure 10. Violin plots of the normalized Euclidean distance (upper panel) and Pearson correlation (bottom panel) between the empirical and simulated Functional Connectivity Dynamics (FCD) and static Functional Connectivity (FC). As explained in the main manuscript text, model optimization was performed using the Euclidean distance as the metric and the empirical FCD as the target. (PCB: Placebo; DMT: dimethyltryptamine).
